# Thoracolumbar Sacral Orthosis for Spinal Fractures: What’s the Evidence and Do Patients Use Them?

**DOI:** 10.7759/cureus.31117

**Published:** 2022-11-05

**Authors:** Sonu Mehta, Baasil S Yusuf, Daphne Chiew, Sameer Rathore, Nallamilli R Reddy, Deepak Nair, Uday Mahajan, Thayur R Madhusudhan, Adhiyaman Vedamurthy

**Affiliations:** 1 Department of Trauma and Orthopaedics, Glan Clwyd Hospital, Rhyl, GBR; 2 Department of Trauma and Orthopaedics, Airedale General Hospital, Keighley, GBR; 3 Department of Trauma and Orthopaedics, Whittington Hospital, London, GBR

**Keywords:** tlso brace, spinal bracing, thoracolumbar spine fracture, thoracolumbar spine orthosis, thoracolumbar fracture

## Abstract

Introduction

The general consensus regarding the non-operative management of thoracolumbar (TL) spine fractures revolves around the use of thoracolumbar spine orthosis (TLSO). The efficacy of TLSO bracing remains controversial within the current literature, with several studies showing that prolonged brace use is associated with diminished lung capacity, skin breakdown, and paraspinal muscular atrophy, with no significant difference in pain and functional outcomes between patients treated with or without TLSO.

Aims

The aim of this study was to identify the number of braces issued over the duration of the study and understand the cost implication, added length of stay, and patient satisfaction based on a questionnaire and reflect on whether we need to change our practice on the use of TLSO.

Methods

Data was collected retrospectively over an 18-month period with 75 patients being initially identified for the study. A total of 42 records were included in the final study after the application of inclusion/exclusion criteria. Patient-related outcomes were recorded through a questionnaire.

Results

Of the patients, 60% report not receiving adequate advice regarding the duration of treatment, 43% reported that the brace interfered with their activities of daily living (ADLs), and 73% came off the brace earlier than advised, with 60% of patients reporting that they would rather be without the brace if given the option. The average increase in length of stay waiting for bracing was three days, with the estimated cost incurred being approximately £114,000.

Conclusion

With equivalence between treatment with or without a brace, there is a need to rethink current practice and move toward a case-by-case, patient-centered approach to minimize costs and improve patient satisfaction.

## Introduction

Thoracolumbar (TL) spine injuries account for the highest incidence of spine injuries [1]. Consequences of TL spine injuries range from mild back pain to deformity, paralysis, and loss of function. Treatment is either surgical fixation or conservative management, and this is determined by the type of fracture, neurological status, and patient comorbidities [2]. The consensus regarding non-operative management involves the use of thoracolumbar spine orthosis (TLSO). TLSO maintains a relative extension locked position, with the aim of reducing the load transferred via the anterior column, limiting segmental motion, subsequently leading to good fracture healing and pain relief. However, several studies have shown that prolonged brace use may lead to diminished pulmonary capacity, skin breakdown, weakness of paraspinal musculature, and no significant difference in pain and functional outcomes between patients treated with or without a brace [3-6].

The purpose of this study was to identify the number of braces issued over the duration of the study and understand the cost implication, added length of stay, and patient satisfaction based on a questionnaire and reflect on whether we need to change our practice on the use of TLSO.

## Materials and methods

**Figure 1:**
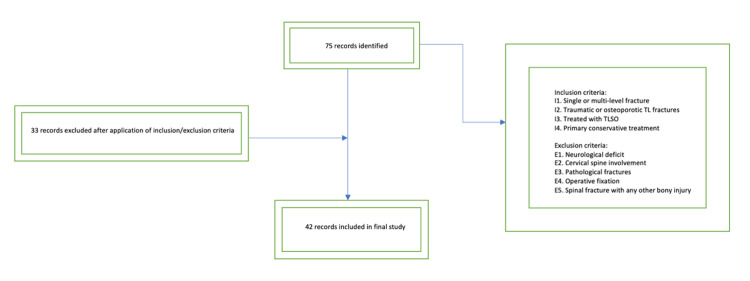
Flowchart displaying patient selection and inclusion/exclusion criteria

This retrospective cohort study took place over an 18-month period from January 2020 to July 2021 in a District General Hospital. Patients were identified from the TLSO issue list provided by the orthotics department. TL fracture diagnosis was confirmed through a review of the relevant radiology (radiograph, computerized tomography, and magnetic resonance imaging), and medical records were reviewed to confirm neurological status at the time of injury and documented non-operative management (Figure 1). The time between when the brace was ordered and the date of discharge was measured. The brace that was used in this study was the Horizon 456 TLSO (Aspen Medical Products, Irvine, CA, USA).

Patient-related outcomes (PROs) were assessed using a questionnaire focusing on practicality, comfort, and impact on activities of daily living (ADLs) (Appendices). Patients were contacted via telephone.

## Results

**Figure 2:**
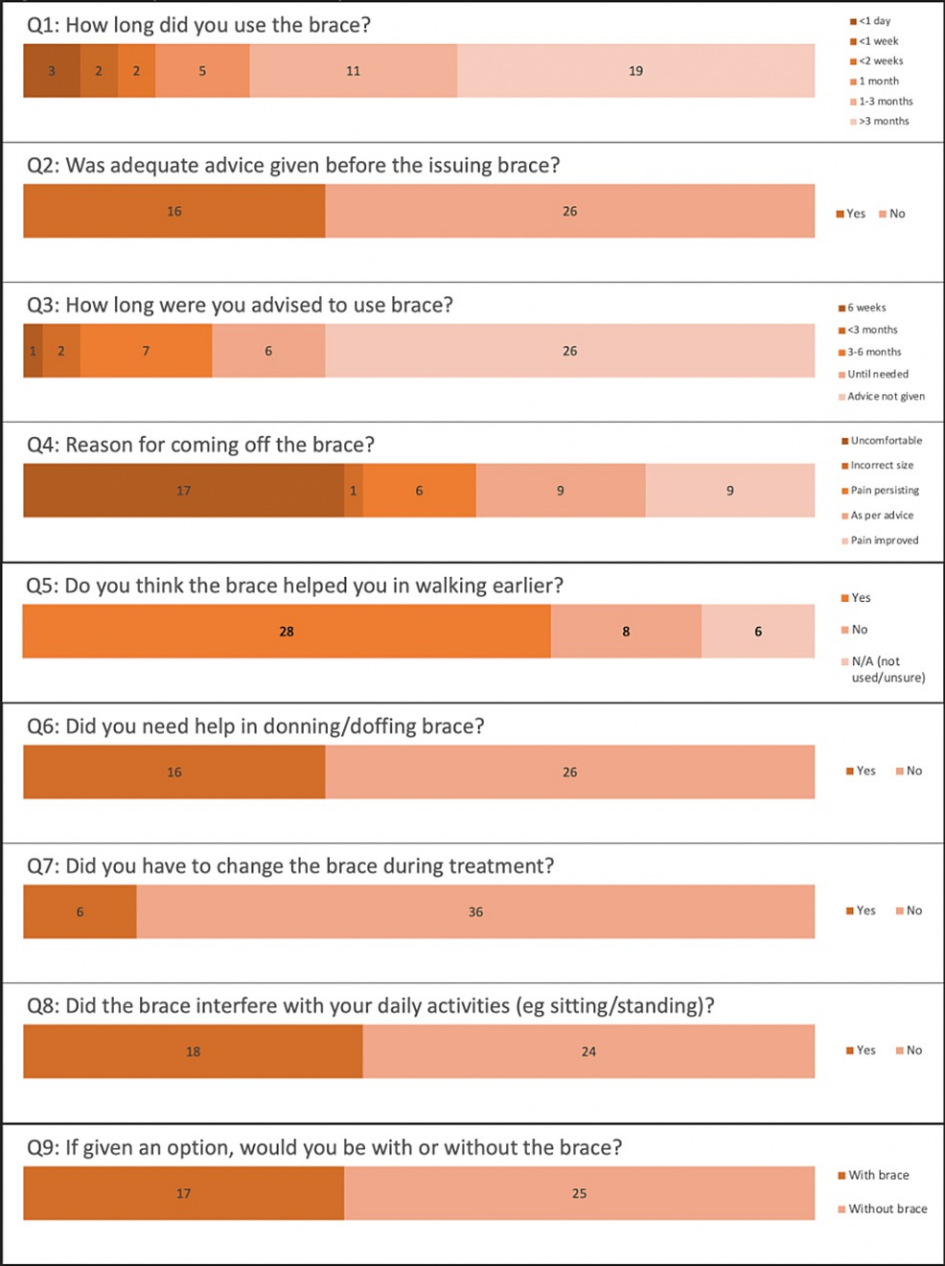
Bar chart displaying questionnaire responses

A total of 72 braces were issued during the timeframe. The mean age of our cohort was 70 years. Forty-two patients remained in the study based on the inclusion/exclusion criteria. Questionnaire responses are highlighted in Figure 2.

Of the patients, 71% wore the brace for over one month, with 45% of those patients wearing it for more than three months. The remaining patients wore the brace for less than one month, with 64% of those patients taking the brace off within two weeks. Only 40% of the patients report being advised on how long to wear the brace at the time of inpatient discharge. Of the patients, 40% discarded the brace due to it being uncomfortable, and 14% stopped wearing the brace as they remained pain-free. Additionally, 65% of the patients felt that the brace was key in allowing them to mobilize earlier. Of the patients, 43% felt that the brace interfered with their activities of daily living (ADLs). If given the option, 60% of the patients reported that they would rather be without the brace. Additionally, 73% of the patients came off the brace earlier than advised. Patients waited an average of three days in the hospital for the TLSO brace prior to discharge, adding to the length of stay.

## Discussion

Although the consensus regarding the non-operative management of TL spine fractures involves TLSO, emerging evidence suggests that there may be little difference between managing stable TL spine fractures with TLSO versus without bracing. The current literature can further be divided between traumatic and non-traumatic (i.e., osteoporotic) TL fractures. A systematic review and meta-analysis conducted by Linhares et al. focused on patients with acute traumatic TL fractures and reported no significant impact of TLSO use in pain, disability, kyphosis progression, or loss of anterior vertebral height when compared with no immobilization [7]. Interestingly, in our study, out of the 73% of patients who came off the brace earlier than advised, 66% of these patients had sustained acute traumatic fractures. While part of the reason may be that they may have not received adequate advice, this may also be explained by the fact that these fractures occurred in a younger population in comparison to the atraumatic fractures; hence, better fracture healing and premorbid status may contribute to these findings.

A systematic review performed by Kweh et al. reviewed the use of orthoses in atraumatic osteoporotic fractures in patients aged 60 years or older. Contrary to the popular belief that TLSO immobilization may lead to muscle atrophy and be paradoxically detrimental to fracture healing and posture, the use of a dynamic semirigid TLSO can generate tactile feedback for the wearer in addition to providing stability, thus serving as a reminder for the user to activate their own musculature and minimize a kyphotic posture through a biomechanical feedback loop [8]. Despite the benefits of enhanced postural stability, pain relief, and improved muscular strength, this study highlights the increased incidence of orthoses-associated complications such as pressure sores, soft tissue infections, and impaired respiratory effort, increasing the risk of lower respiratory tract infections [8].

Several studies reported no significant difference in pain scores between patients managed with and without a brace [9-12]. Interestingly, 40% of the patients came off the brace earlier than advised due to the discomfort associated with the brace, and a further 13% came off the brace due to pain persisting. Of the patients, 43% reported that bracing negatively impacted their quality of life, despite 67% of patients reporting that they felt the TLSO allowed them to walk earlier. Hoshino et al. performed a prospective cohort study using 36-Item Short Form Survey (SF-36) scores and identified no statistically significant difference in the quality of life between bracing and no bracing [13]. Conversely, Pfeifer et al. reported a statistically significant improvement in the quality of life with patients using TLSO compared to non-bracing using Hobi’s scale as a quality of life indicator [14].

Two studies looked at the psychological benefits of orthosis use. Kato et al. and Hoshino et al. identified an improvement in mental health irrespective of brace use [13,15]. Our study showed that 67% of the patients psychologically benefitted from bracing and felt that it enabled early mobilization. Remarkably, 70% of these patients were aged 70 or above.

Linhares et al. estimated that the length of stay was 3.47 days higher in patients waiting for orthosis [8]. Our study showed a similar length of stay with patients waiting for an average of three days from the date the brace was ordered to the date of discharge.

Considering the average cost of one TLSO being approximately £200 and the three-day extended stay in the hospital costing approximately £1,320, we conservatively estimated that the cost incurred by our hospital over this 18-month period would be around £114,000.

Limitations of this study include its retrospective nature with the absence of a control group and a small sample size. Additionally, increased length of hospital stay may be multifactorial, such as patients awaiting a package of care or new discharge destination.

## Conclusions

TLSO remains a key part of the non-operative management of TL fractures. Current studies suggest that bracing may be more effective in atraumatic fractures than in acute traumatic fractures. Our study not only corroborates this but also highlights the importance of giving patients clear advice on proper usage, time in brace, and managing ideas, concerns, and expectations regarding pain and mobility. Significant cost is associated with widely distributing braces, and we propose a case-by-case approach to minimize costs and improve patient satisfaction. If there is an equivalence between treatment with and without a brace, there is a need to rethink current practice through further research.

## Appendices

The questionnaire used to assess patient-related outcomes (PROs) is shown in Table 1.

**Table 1:** Patient feedback questionnaire TLSO: thoracolumbar spine orthosis

TLSO Patient Feedback Questionnaire	
1. How long did you use the brace?	<1 day, <1 week, <2 weeks, 1 month, 1-3 months, >3 months
2. Was adequate advice given before issuing the brace?	Yes/no
3. How long were you advised to use the brace?	6 weeks, <3 months, 3-6 months, until needed, advice not given
4. Any specific reason for coming off the brace earlier?	Uncomfortable, incorrect size, pain persisting, as per advice, pain improved
5. Do you think the brace helped you in walking earlier?	Yes/no/NA (not used/unsure)
6. Did you need help in donning/doffing the brace?	Yes/no
7. Did you have to change the brace during treatment?	Yes/no
8. Did the brace interfere with your daily activities like sitting, standing, etc.?	Yes/no
9. If given an option, would you be with or without the brace?	With brace/without brace
Any other comments in relation to the brace	
